# Guidelines for otorhinolaryngologists and head neck surgeons in coronavirus disease 2019 pandemic

**DOI:** 10.1186/s43163-021-00082-0

**Published:** 2021-02-17

**Authors:** Meenesh Juvekar, Baisali Sarkar

**Affiliations:** 1 Department of Otorhinolaryngology, Grant Medical College and J.J Group of Hospitals, Mumbai and Bombay Hospital and Research Centre, Mumbai, Maharashtra India; 2 Department of Otorhinolaryngology, Guwahati Neurological Research Centre, Kolkata, West Bengal India

**Keywords:** SARS-CoV 2, COVID-19, Pandemic, Otorhinolaryngologist, Guidelines

## Abstract

**Background:**

Coronavirus disease 2019 was first identified in Wuhan, the capital of China’s Hubei province, in December 2019. India has witnessed a massive surge of coronavirus cases.

**Main text:**

This study details the measures to be taken by the clinicians involved in doing otorhinolaryngology and head neck surgery in light of the recent coronavirus disease 2019 pandemic. All COVID-positive patients should be admitted in a separate COVID ward, and patients should be screened for COVID-19 before admission. Only emergent ENT surgeries should be done in an operating room having a negative pressure environment with high-frequency air changes, and all staff must wear personal protective equipment. The anesthetist intubates the patient while the surgical team waits outside the operation theater post-intubation for 21 min. For otology surgery, double draping of the microscope should be done; for rhinology surgery, concept of negative-pressure otolaryngology viral isolation drape (NOVID) system should be used. Smoke evacuation system is set up inside the tent to evacuate any smoke produced during the surgery. Tracheostomy should be done at least after 10 days of mechanical ventilation with cuffed, non-fenestrated tracheal tube inserted through the tracheal window, and a separate closed suction system is used for suctioning. After the surgery is completed, disposal of PPE kit needs to be done according to local guidelines. After completion of the surgery, the full anesthesia unit should be disinfected for 2 h with 12 % hydrogen peroxide. Chlorine-containing disinfectant (2000 mg/L) is used to clean the floor of the operation theater and clean all the reusable medical equipment. Ultra-low volume 20 to 30 mL/m of 3% hydrogen peroxide is used to fumigate the OT for 2 h.

**Conclusions:**

COVID-19 is a newly discovered infectious disease. Measures need to be taken to prevent transmission and attain a plateau and decline in the disease. Otorhinolaryngologists and head neck surgeons are at high risk of this infection. This review summarizes the protocol for otorhinolaryngologists and head neck surgeons caring for patients in this current scenario. Protocols need to be strictly followed to prevent the spread of this disease.

## Background

Severe acute respiratory syndrome coronavirus 2 (SARS-CoV-2) is the causative agent for coronavirus disease 2019 (COVID-19) [1]. COVID-19 disease was first identified in December 2019 in Wuhan, the capital of China’s Hubei province, and it caused a worldwide pandemic [2, 3]. Coronavirus causes respiratory tract infections, the severity of which can be mild, like common cold, and can be lethal, like SARS (severe acute respiratory syndrome) and MERS (Middle East respiratory syndrome). The virus is most contagious when people are symptomatic, although spread is possible even before symptoms appear [4]. The virus mainly spread by close contact via small droplet produced while coughing, sneezing, and talking [4, 5]. Bioaerosol transmission occurs while doing intubation and cardiopulmonary resuscitation, and even fomite transmission is also possible [4, 6]. The virus survives for hours to days on surfaces. The patient may be asymptomatic or present with flu-like symptoms like fever, cough, sneezing, fatigue, and shortness of breath. The disease may progress to pneumonia, multi-organ failure, and even death [7–10]. Olfactory dysfunction is currently the most common clinical feature of COVID-19 [11]. Anosmia is the most distinguished sign of COVID-19 disease with the patient may present with anosmia of sudden onset [12]. The incubation period is 5 to 6 days but may range from 2 to 14 days. ENT, head and neck surgeons, and health care staff are at high risk of COVID-19 infection; hence, appropriate protective and hygiene measures are of utmost importance [13]. The risks seem to be more high in the field of rhinology and endoscopic endonasal surgery [14]. Genuine concerns are raised for nasal endoscopy, flexible laryngoscopic examination of patients in outpatient clinics and during surgery, as virus reside primarily in the nasal cavity and nasopharynx.

## Main text

### Data sources

Protocols relating to otolaryngology practice were identified from webpages of otolaryngology societies such as the American Academy of Otolaryngology-Head and Neck Surgery and American Head and Neck Society [15]; French scientific societies: the French ENT Society, French Rhinology Association, French ENT College, French ENT National Union, and French National Professional ENT Council [16]; Centers for Disease Control and Prevention; Web of Science; PubMed; and Google Scholar.

### Protocol for ENT and head neck procedures

1. ENT and head and neck surgical ward

- There should be COVID-free ward and a separate COVID ward for COVID negative and positive or suspected patient, respectively.
- Patients should be screened for COVID-19 before admission.
- Distancing of at least 2 ms in between patient beds is mandatory.
- Ward should be demarcated into a separate area for patients with high aerosol generating potential.
- Only one care-taker should be allowed at a time who is also screened like above and they should comply to strict precaution for COVID 19.
- Appropriate hand washing and hand-hygiene supplies should be ensured.
- Examination instruments should be properly sterilized after every use.
- Ward should have minimum furniture.
- Visitors should not be allowed.
- Corridors and rooms should be well-ventilated.
- All hospitals and clinics should maintain proper biosafety and precautions because there are many patients who are infected but asymptomatic yet have potential risk to spread disease.

2. Decision and timing for procedures [17]

- When a patient comes for ENT and head and neck surgery, firstly, the surgery is graded as:

i. *Emergent surgery* **-** It means one that needs immediate surgical intervention.
ii. *Urgent surgery* **-** It means delay of treatment for 1 month may harm the patient, and
iii. *Elective routine* **-** These surgeries are preferably deferred till the COVID-19 pandemic gets over.

3. Place of procedure [18]

- Proper operating room is preferred for surgical intervention.
- The operating room should have negative pressure environment with high-frequency air changes (25 per hour).
- Each operating room should have a separate ventilating system with integrated high-efficiency particulate air (HEPA) filter.
- Separate operating rooms should be designated for COVID-19 suspected or positive patients.
- There should be a negative pressure isolation transfer cabin which is to be used to transfer patients between the isolation ward and operation theater. Those who are involved must wear level 3 protective medical equipment (which means protection of mouth, nose, eyes, body, and hand).
- Principles of clean area, contaminated pollution area, and two buffer zones should be followed while entry and exit into the operating theater.

4. Staff preparation

- All staff must wear personal protective equipment (PPE) like cap, powered air purifying respirator (PAPR), eye protection, face shield, fluid-repellent disposable surgical gown, double gloves, and shoe cover [19–23].
- If PAPR is not available, then FFP3 mask (fit-tested filtering facepiece 3) or N95 mask is used with an additional fluid shield.
- Staff are designated to form a core COVID airway team to help facilitate efficiency during the crisis.
- Limit the attendance to essential personnel inside the operation theater.
- Staff who are 55 years and above, pregnancy, chronic diseases like diabetes mellitus, cancer, renal diseases, chronic hepatitis, and autoimmune diseases are associated with risk factors for developing severe acute respiratory distress syndrome. Thus, health workers who are suffering from any ailment like these should not involve in nursing and treating COVID-19 patients [24].
- It is mandatory to isolate and observe healthcare personals working in COVID-19 suspicious or infected patients if they come in close unprotected contact with COVID-19 pneumonia patients or they show symptoms of COVID-19 infection or when work in COVID-19 infection ward is finished.
- Nasopharyngeal or oropharyngeal swabs for COVID-19 need to be done.
- Those tested positive should undergo strict isolation and observation, while others isolated for observation and start work after 1 week [25].

5. Procedure

- The procedures of ENT and head and neck have high possibility to aerosolize aerodigestive secretions like endotracheal intubation. Non-invasive ventilation, naso-laryngoscopy, transnasal endoscopic surgery, and ultrasonic instruments or high-speed handpieces escalate the risk of COVID-19 infection, and thus, it should be done only when it is mandatory [26, 27].
- All *Emergent cases* need to obey standard COVID-19 protocols even if the patients’ COVID status is unknown or even positive.
- Anesthetist intubates the patient while the surgical team waits outside the operation theater post-intubation for 21 min [28].
- After proper dressing and draping, transparent plastic drape is used to cover the patient to prevent viral spread from the ear, nose, nasopharynx, and endotracheal tube into the environment.
- Procedure in otology surgery [28]-

**Fig. 1:**
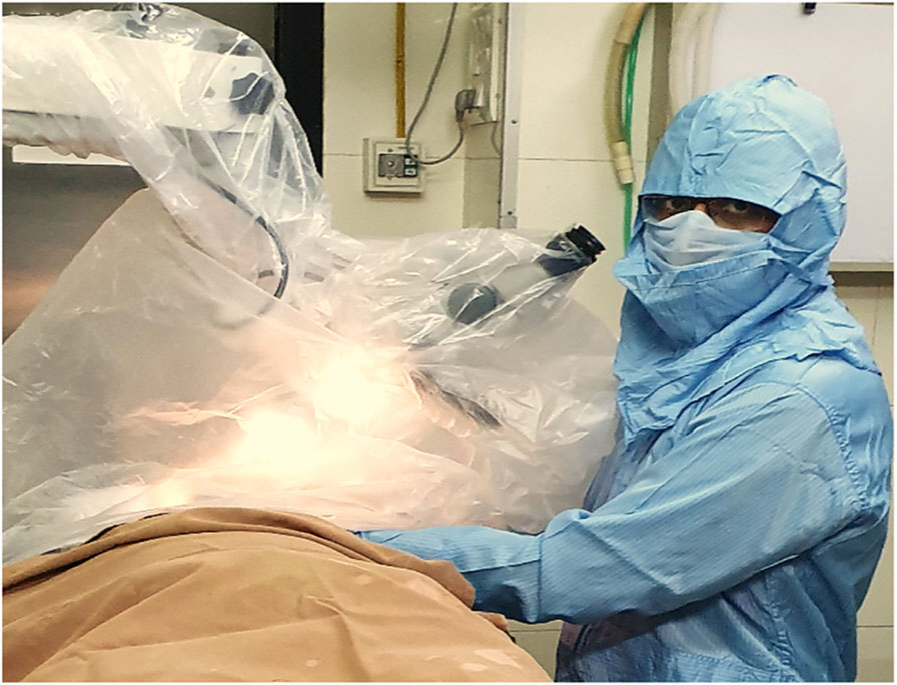
Plastic drape used to make tent and drape the patient while doing otology surgery

➢ The microscope is draped normally, then a second drape is attached to the lens and extended over the head of the bed to create a plastic tent (Fig. 1).

➢ Smoke evacuation system is set up inside the tent.

➢ If mastoidectomy or drilling is needed, then it is done within the tent to reduce aerosol reaching the environment.

➢ Suction is done through the caudal aspect of the plastic tent.

- Procedure in rhinology surgery [29]:

➢ Transparent plastic drape also called negative-pressure otolaryngology viral isolation drape (NOVID) system 112 cm × 112 cm fluid warmer drape is kept over Lone Star disposable ring retractor and is suspended by a Bookwalter Retractor Laparoscopic Support Set; this produces a tent and prevents the plastic drape to collapse on instruments while doing surgery [30].

➢ In Indian scenario, if the above mentioned NOVID is not available, then a plain transparent plastic drape is used to cover the patient and the drape is placed on a disposable ring-shaped material and a retractor is used to retract the whole unit, thus creating a closed chamber.

➢ The transparent plastic drape forms a compact chamber; the end of the plastic drape is attached to the patient’s bed by towel clips.

➢ Smoke evacuation system is set up inside the tent to evacuate any smoke produced during the surgery.

➢ If endoscope, microdebrider, or drilling is needed, then it is done through small fenestrations made on the transparent plastic drape to reduce aerosol reaching the environment, and suction is also done through the caudal aspect of the transparent plastic drape.

- In larynx, procedures like tracheostomy are high aerosol generating procedures. Thus, meticulously planned decisions are taken to do the surgery and certain special steps done to reduce spread of infection [31, 32]:

➢ Decision for timing for tracheostomy is at least after 10 days of mechanical ventilation.

➢ Two ICU consultants should make a decision after discussion with the surgical team and senior anesthetist.

➢ The patient should have a good expectation of achieving complete recovery and having an independent lifestyle.

➢ It is very important to inform the anesthetist of readiness to open trachea.

➢ Confirm paralysis and request full paralysis throughout to reduce risk of cough.

➢ Pre-oxygenate with PEEP (positive end-expiratory pressure) is done then stop ventilation and turn off flows.

➢ Allow time for passive expiration with open APL (adjustable pressure limiting) valve.

➢ Consider clamping of the ETT then advance cuff beyond the proposed tracheal window.

➢ Hyperinflate cuff and re-establish oxygenation with PEEP.

➢ When adequately oxygenated, communicate clearly and cease ventilation prior to opening of the trachea. This reduces the duration of apnea.

➢ Create tracheal window taking care to avoid the ETT cuff injury.

➢ Turn off flows with open APL valve, allow passive expiration, consider clamping ETT.

➢ Deflate ETT cuff and draw back proximal to the tracheal window under direct vision.

➢ Ensure tracheal window is of sufficient size to allow easy insertion of tracheostomy tube without injury to cuff.

➢ Insert cuffed, non-fenestrated tracheal tube through the tracheal window.

➢ Immediately inflate the tracheostomy tube cuff.

➢ Replace introducer with non-fenestrated inner tube and HME (heat and moisture exchange) filter.

➢ Prompt attachment of circuit is done.

➢ Resume ventilation immediately.

- After closure of the surgical wound, first, the transparent plastic drape is removed and then the patient drape is carefully rolled and removed.
- Thereafter, the patient is cleaned and the anesthetist extubates the patient while the surgical team waits outside the operation theater post-extubation for 21 min.

6. Proper disposal of equipment and decontamination of operation theater [31]

- After surgery is completed, disposal of PPE kit needs to be done according to local guidelines.
- After completion of surgery, the full anesthesia unit should be disinfected for 2 h with 12% hydrogen peroxide present inside an anesthesia circuit sterilizer.
- Chlorine-containing disinfectant (2000 mg/L) is used to clean the floor of the operation theater and clean all the reusable medical equipment.
- Surgical instruments are soaked in 2000 mg/L chlorine-containing disinfectant and then sealed in double-layer disposable waste bags and sent to the respective disinfection area.
- The air purification system of the operation theater is shut down after 30 min and then an ultra-low volume 20 to 30 mL/m of 3% hydrogen peroxide is used to fumigate the OT for 2 h, after which the negative pressure ventilation is turned on again.

7. In Urgent cases, the protocol is as follows:

- The patient and family member are asked to self-quarantine till the surgery.
- The patient is contacted 5 days before the surgery and a date and time for COVID-19 testing is informed.
- Forty-eight to 72 h prior to surgery, nasopharyngeal swab is taken and send for real-time reverse transcription polymerase chain reaction (rRT-PCR) for COVID-19.
- If the initial test is negative, then the test is repeated within 24 h prior to surgery, and HRCT of the chest is done to exclude any false negative test result.
- HRCT chest positive findings include ground-glass opacity, local patchy shadowing, bilateral patchy shadowing, or interstitial abnormalities [33].
- If the tests are negative, then standard COVID-19 protocol is followed.
- If the initial test is positive or the repeat test is positive, then it is better to defer the case and try alternative treatment.
- If the case cannot be deferred, then standard COVID-19 protocol is maintained while doing the operation.

8. *Elective routine* surgeries are preferably deferred till the COVID-19 pandemic gets over.

## Conclusion

In humans, it has been found that there are 7 types of coronaviruses namely SARS-CoV-2, severe acute respiratory syndrome coronavirus (SARS-CoV), Middle East respiratory syndrome coronavirus (MERS-CoV), HCoV-NL63, HCoV-OC43, HCoV-229E, and HCoV-HKU1 [34]. SARS-CoV-2 genome is a 29,903-bp single-stranded RNA coronavirus [35]. SARS-CoV-2 virus bears a spiny protein named S1 which adhere to the ACE2 receptor present on the host cell membrane [34]. Even though data on infectivity of SARS-CoV-2 is very less, infection and death amid health-care staff have been reported [36, 37]. The incubation period of SARS-CoV-2 is approximately 5 days (range 4–14 days) [38, 39]. SARS-CoV-2 is normally profuse during the onset of the symptom, after which the viral load typically reduces following 3–4 days [40]. In most patients, PCR samples for SARS-CoV-2 taken from the lower respiratory tract is positive up to 39 days [41]. The antiviral antibody typically appears both in the blood and respiratory secretions in around 7 days after onset of symptom, and in 90% of the patients, anti-viral antibody is detectable by 12 days after onset of symptom [42]. The presence of anti-viral antibody impedes the infectivity of detectable virus. The procedures of ENT and head and neck have high possibility to aerosolize aerodigestive secretions.

If strict safety guidelines are followed, then the probability to get COVID-19 infection reduced. Whenever any patient comes for ENT procedure, COVID-19 test is must. Any suspected or positive patient is kept in a separate COVID ward. Strict guidelines for social distancing, hand washing, hygiene, ventilation, and avoiding visitors must be followed. Decision for surgery is crucial and is taken by a senior surgeon and anesthetist. Although surgical cases are deferred, still crucial life-threatening cases need to get operated. High-speed handpiece microdrills escalate the risk of COVID-19 infection, and thus, it should be used only when it is mandatory [17, 18]. Specific masks like N95 or FFP2 or powered air purifying respirator (PAPR) should be used. PPE kits and firm sterilization measures should be used to circumvent COVID-19 infection [19, 20]. Protection is taken while transferring the patient to the operation room. The operating room should have a negative pressure environment with HEPA filter. Double draping is beneficial and reduces the spread of aerosol. Proper sanitization of the operation room and equipment reduces the viral load to a great extent. It is more effective to keep highly aerosol-generating post-operative patients like post-tracheostomy patients in an isolated ward; this will safeguard other patients and healthcare personals.

It is yet uncertain about the resolution of COVID-19 disease and when serology testing will become more extensive, or when therapeutics and a vaccine will be available. With the case count rising worldwide, doctors need to adopt best care practices and learnings from the experiences of colleagues from across the world to safeguard themselves and in turn, their patients and family.

COVID-19 disease being highly contagious, measures need to be taken to prevent the transmission of the disease and thus attaining a plateau and decline in the disease. Otolaryngologists, dentist, and ICU doctors hold an important position who see patients with manifestations of COVID-19 and have a significantly higher risk of infection. This review summarizes the protocol for ENT and head neck surgeons caring for patients in this current scenario and serves as a template to structure their practices in the current outbreak. New practices and suggestions will evolve in ushering days based on new data and availability of testing and resources. Protocols need to be strictly followed to prevent the spread of this disease.

## Data Availability

Protocols relating to otolaryngology practice were identified from webpages of otorhinolaryngology societies such as the American Academy of Otolaryngology-Head and Neck Surgery and American Head and Neck Society; French scientific societies: the French ENT Society, French Rhinology Association, French ENT College, French ENT National Union, and French National Professional ENT Council; Centers for Disease Control and Prevention; Web of Science; PubMed; and Google Scholar.

## Abbreviations

SARS-CoV-2: Severe acute respiratory syndrome coronavirus 2
COVID-19: Coronavirus disease 2019
SARS: Severe acute respiratory syndrome
MERS: Middle East respiratory syndrome
rRT-PCR: Real-time reverse transcription polymerase chain reaction
ENT: Ear, nose, throat
OPD: Outpatient department
HEPA: High-efficiency particulate air
PPE: Personal protective equipment
PAPR: Powered air purifying respirator
FFP3: Fit-tested filtering facepiece 3
NOVID: Negative-pressure otolaryngology viral isolation drape
ICU: Intensive care unit
PEEP: Positive end-expiratory pressure
APL: Adjustable pressure limiting
ETT: Endotracheal tube
HME: Heat and moisture exchange
HRCT: High-resolution computed tomography
